# Characterization of Essential Oil Composition in Different Basil Species and Pot Cultures by a GC-MS Method

**DOI:** 10.3390/molecules22071221

**Published:** 2017-07-20

**Authors:** Andrea Muráriková, Anton Ťažký, Jarmila Neugebauerová, Alexandra Planková, Josef Jampílek, Pavel Mučaji, Peter Mikuš

**Affiliations:** 1 Department of Vegetable Growing and Floriculture, Faculty of Horticulture, Mendel University in Brno, Valtická 337, 691 44 Lednice, Czech Republic; xmurarik@node.mendelu.cz (A.M.); jarmila.neugebauerova@mendelu.cz (J.N.); 2 Department of Pharmaceutical Analysis and Nuclear Pharmacy, Faculty of Pharmacy, Comenius University in Bratislava, Odbojárov 10, SK-832 32 Bratislava, Slovak Republic; tazky@fpharm.uniba.sk (A.Ť.); plankova@fpharm.uniba.sk (A.P.); 3 Toxicological and Antidoping center, Faculty of Pharmacy, Comenius University in Bratislava, Odbojárov 10, SK-832 32 Bratislava, Slovak Republic; 4 Department of Pharmaceutical Chemistry, Faculty of Pharmacy, Comenius University in Bratislava, Odbojárov 10, SK-832 32 Bratislava, Slovak Republic; josef.jampilek@gmail.com; 5 Department of Pharmacognosy and Botany, Faculty of Pharmacy, Comenius University in Bratislava, Odbojárov 10, SK-832 32 Bratislava, Slovak Republic; mucaji@fpharm.uniba.sk

**Keywords:** essential oils, basil varieties, pot cultures, profiling, GC-MS analysis

## Abstract

Basil (*Ocimum* L.) species are used as medicinal plants due to their essential oils exhibiting specific biological activity. The present work demonstrated that both the variety and season/conditions of cultivation had a significant effect on (i) the produced amount (extraction yield), (ii) qualitative, as well as (iii) quantitative profile of basil essential oil. Among studied basil varieties, a new variety, ‘Mánes’, was characterized for the first time. Based on our quantitative evaluation of GC-MS profiles, the following chemotypes and average concentrations of a main component were detected in the studied basil varieties: ‘Ohře’, ‘Lettuce Leaf’, ‘Purple Opaal’, ‘Dark Green’ (linalool, 5.99, 2.49, 2.34, 2.01 mg/mL, respectively), and ‘Mammolo Genovese’, ‘Mánes’, ‘Red Rubin’ (eucalyptol, 1.34, 0.96, 0.76 mg/mL, respectively). At the same time, when considering other compounds identified in GC-MS profiles, all the studied varieties, except from ‘Lettuce Leaf’, were methyl eugenol-rich with a strong dependence of the eugenol:methyl eugenol ratio on the seasonal changes (mainly solar irradiation, but also temperature and relative humidity). More complex and/or variable (depending on the season and cultivation) chemotypes were observed with ‘Lettuce Leaf’ (plus estragole, 2.27 mg/mL), ‘Dark Green’ (plus eucalyptol, 1.36 mg/mL), ‘Mammolo Genovese’ (plus eugenol, 1.19 mg/mL), ‘Red Rubin’ (plus linalool and eugenol, 0.46 and 0.56 mg/mL, respectively), and ‘Mánes’ (plus linalool and eugenol, 0.58 and 0.40 mg/mL, respectively). When considering superior extraction yield (ca. 17 mL·kg^−1^, i.e., two to five times higher than other examined varieties) and consistent amounts (yields) of essential oil when comparing inter-seasonal or inter-year data (RSD and inter-year difference in mean yield values ˂2.5%), this new basil variety is very promising for use in the pharmaceutical, food, and cosmetic industries.

## 1. Introduction

Basil (*Ocimum* L.) species have been used for centuries as medicinal plants. Basil essential oils are responsible for characteristic aroma and biological activity [1,2]. The chemical composition of sweet basil essential oil, similarly to other oil plants, depends on genetic, ontogenetic, and environmental factors [3]. Hence, the morphological and chemical variability of the basil creates great possibilities for growing different cultivars of this valuable herbal plant. Numerous basil cultivars and forms, differing in the plant size, habitus, color, and shape and size of the leaves and flowers [4], content and chemical composition of the essential oil [5,6,7], as well as other biologically active substances [8], are currently cultivated in Europe and in the world. 

Lawrence et al. [9] classified four major chemotypes of basil based on essential oil composition: (1) methyl chavicol (estragole)-rich; (2) linalool-rich; (3) methyl eugenol-rich; (4) methyl cinnamate-rich, and also numerous subtypes. According to the geographical origin of basil varieties and on the basis of their major constituents, they are classified in four chemotypes [10]: (1) European chemotype, the oil of which is characterized by high amounts of linalool (35–50%) and estragole (15–25%); (2) reunion chemotype (estragole basil) whose main essential oil component is estragole (80% or more); (3) tropical chemotype (cinnamon basil), the oil of which is dominated by methyl cinnamate, and (4) eugenol chemotype whose major oil component is eugenol.

The qualitative and quantitative profiles of particular basil varieties can differ significantly depending on their geographical origin as it can be seen, for example, for the ‘Purple Opaal’ variety [11,12] or the ‘Red Rubin’ variety [13,14]. Linalool is the dominant component of the oil derived from European basil varieties [11,13,15,16]. In addition, the components that occurred in larger amounts were geraniol (12.6%), 1.8-cineole (4.1%), and epi-α-cadinol (3.8%). In other studies, the major compounds in basil oil reported were linalool, methyl chavicol, eugenol, bergamotene, and methyl cinnamate [12], or methylchavicol, camphor, citral, limonene, methylcinnamate, caryophyllen-β, anethol, terpinen-4-ol, myrcene, thymol, ocimene, and cinnamaldehyde [17]. Chalchat and Özcan [18] showed different concentrations of linalool and other important components in the basil essential oil extracted from the different parts of the herb, such as flowers, leaves, and stems. Among the main constituents of flower oil were estragole (58.26%), fenchone (10.1%), limonene + β-phellandrene (19.41%), and α-phellandrene (4.37%), while estragole (52.60%), limonene (13.64%), exo-fenchyl acetate (10.99%), fenchone (5.70%), α-phellandrene (4.15%), and endo-fenchyl acetate (1.30%) were found as the main components of basil leaf oil. Recently, the effect of elicitation with jasmonic acid (JA) on the plant yield, the production and composition of essential oils of 'Lettuce leaf' basil was evaluated [19].

The continual increase of the production of basil essential oil reflects in demands on the quantity of basil vegetable matter useful for the extraction. Therefore, great effort is devoted not only to the evaluation of new potent basil varieties, but also to the maximization of periodical harvests (cycles) of the basil plants with acceptable content of essential oil. However, only very few works have been published on the study of seasonal variation of basil oil yield during the year [20]. Valuable European basil varieties such as ‘Ohře’, ‘Lettuce Leaf’, ‘Purple Opaal’, ‘Dark Green’, ‘Mammolo Genovese’, ‘Red Rubin’, and new variety ‘Mánes’, have not been characterized in this manner.

The main objectives of our work, in this context, were (i) to characterize for the first time essential oil yield and chemical profile of a new basil variety, namely ‘Mánes’; (ii) to compare qualitative and quantitative composition and extraction yields of seven sweet basil varieties including ‘Mánes’; and (iii) to evaluate an influence of greenhouse conditions of the cultivation in different seasons of one year on the extraction yield and chemical profile of essential oil of seven sweet basil varieties including ‘Mánes’.

## 2. Results

### 2.1. Evaluation of Extraction Yield of Ocimum Basilicum Varieties

The content of the basil essential oil in the aerial part of plant of seven basil varieties was determined by the method of steam distillation according to [21], without using xylene. The contents of the extracted essential oils obtained from the ‘Ohře’, ‘Lettuce Leaf’, ‘Purple Opaal’, ‘Dark Green’, ‘Mammolo Genovese’, ‘Mánes’, and ‘Red Rubin’ varieties are shown in Table 1. Mean yields for the 3 tested pot cultures ranged from 4.0 to 17.3 (mL·kg^−1^) d. w. (dry weight) in 2015. Similarly, the year 2016 showed the values ranging from 3.7 to 17.7 (mL·kg^−1^) d. w.

Statistical evaluation of the extraction yields obtained from particular varieties and in different years was carried out by means of Tukey’s test. The data presented in Table 1 indicate that as variety as year of cultivation have significant effect on the content (extraction yield) of essential oil.

The most significant statistical difference, with the highest extraction yield, was observed in case of the new variety ‘Mánes’ compared to the other varieties. ‘Mánes’ provided not only the highest extraction yield but also constant/consistent yields of essential oil over all the pot cultures (seasons) studied. These characteristics make the ‘Mánes’ variety very promising for the use in the pharmaceutical, food, and cosmetic industries.

### 2.2. Identification of Essential Oil Components in Ocimum Basilicum Varieties

**Figure 1:**
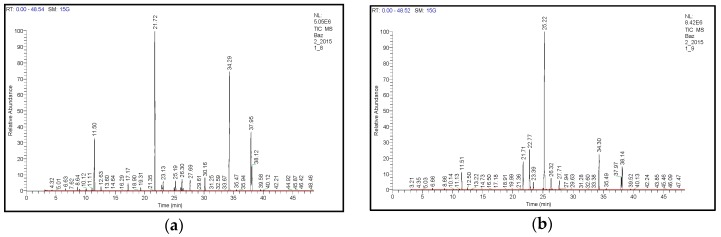

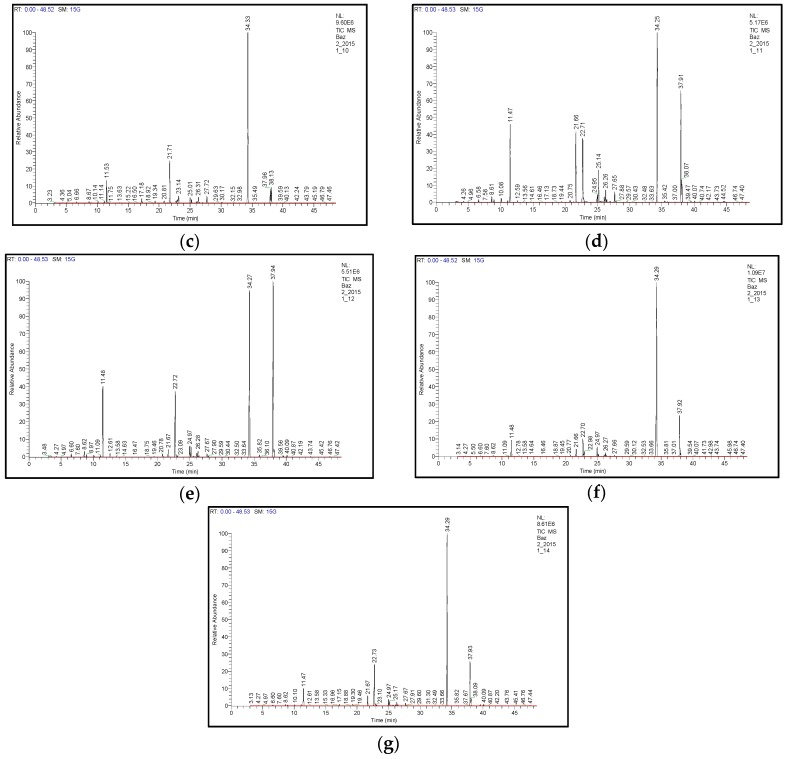
GC-MS profiles illustrating basil essential oil constituents for different basil varieties. (**a**) Ohře; (**b**) Lettuce Leaf; (**c**) Purple Opaal; (**d**) Dark Green; (**e**) Mammolo Genovese; (**f**) Mánes; (**g**) Red Rubin. Analyzed samples corresponded with the pot-culture season No. 2 (summer) and year 2015. For the sample preparation (steam distillation of basil essential oil from plant material) see Section 3.3. For the GC-MS analytical conditions see Section 3.5. Particular components of basil essential oils were identified by means of Thermo Finnigan NIST 02 Libraries software. Eucalyptol (**a**) 11.50 min, (**b**) 11.51 min, (**c**) 11.53 min, (**d**) 11.47 min, (**e**) 11.48 min, (**f**) 11.48 min, (**g**) 11.47 min; Fenchone (**a**) 17.17 min, (**b**) -, (**c**) 17.18 min, (**d**) -, (**e**) -, (**f**) -, (**g**) 17.15 min; Fenchyl acetate (**a**) 19.31 min, (**b**) -, (**c**) 19.34 min, (**d**) -, (**e**) -, (**f**) -, (**g**) 19.30 min; β-Linalool (**a**) 21.72 min, (**b**) 21.71 min, (**c**) 21.71 min, (**d**) 21.66 min, (**e**) 21.67 min, (**f**) 21.66 min, (**g**) 21.67 min; α-Bergamotene (**a**) 22.71 min, (**b**) 22.77 min, (**c**) 22.72 min, (**d**) 22.71 min, (**e**) 22.72 min, (**f**) 22.70 min, (**g**) 22.73 min; Caryophyllene (**a**) 22.88 min, (**b**) -, (**c**) 22.90 min, (**d**) -, (**e**) -, (**f**) 22.98 min, (**g**) -; Isocaryophyllene (**a**) 23.13 min, (**b**) 23.14 min, (**c**) 23.14 min, (**d**) 23.09 min, (**e**) 23.10 min, (**f**) -, (**g**) 23.11 min; 4-Carvomenthol (**a**) -, (**b**) 23.39 min, (**c**) -, (**d**) 23.33 min, (**e**) -, (**f**) -, (**g**) -; β-Farnesene (**a**) 24.98 min, (**b**) 25.01 min, (**c**) 25.01 min, (**d**) 24.95 min, (**e**) 24.97 min, (**f**) 24.97 min, (**g**) -; α-Caryophyllene (**a**) 25.19 min, (**b**) -, (**c**) 25.22 min, (**d**) -, (**e**) 25.18 min, (**f**) 25.17 min, (**g**) 25.17 min; Estragole (**a**) -, (**b**) 25.22 min, (**c**) -, (**d**) 25.14 min, (**e**) -, (**f**) -, (**g**) -; β-Cubebene (**a**) 26.30 min, (**b**) 26.32 min, (**c**) 26.31 min, (**d**) 26.26 min, (**e**) 26.28 min, (**f**) 26.27 min, (**g**) 26.28 min; α-Bulnesene (**a**) 26.48min, (**b**) 26.49 min, (**c**) 26.50 min, (**d**) 26.43 min, (**e**) 26.46 min, (**f**) 26.45 min, (**g**) 26.46 min; γ-Cadinene (**a**) 27.69 min, (**b**) 27.71 min, (**c**) 27.72 min, (**d**) 27.65 min, (**e**) 27.67 min, (**f**) 27.66 min, (**g**) 27.67 min; trans-Geraniol (**a**) 30.16 min, (**b**) -, (**c**) 30.17 min, (**d**) -, (**e**) -, (**f**) -, (**g**) -; Methyl eugenol (**a**) 34.29 min, (**b**) 34.30 min, (**c**) 34.33 min, (**d**) 34.25 min, (**e**) 34.27 min, (**f**) 34.29 min, (**g**) 34.29 min; Eugenol (**a**) 37.95 min, (**b**) 37.97min, (**c**) 37.96min, (**d**) 37.91min, (**e**) 37.94 min, (**f**) 37.92 min, (**g**) 37.93 min; δ-Cadinene (**a**) 38.12 min, (**b**) -, (**c**) 38.13 min, (**d**) 38.07 min, (**e**) -, (**f**) -, (**g**) -.

Separation and identification of particular essential oil components in the seven studied basil varieties was carried out by means of the GC-MS method developed for this purpose (for the optimum GC-MS conditions see Section 3.5), see the samples profiles in Figure 1.

**Figure 2:**
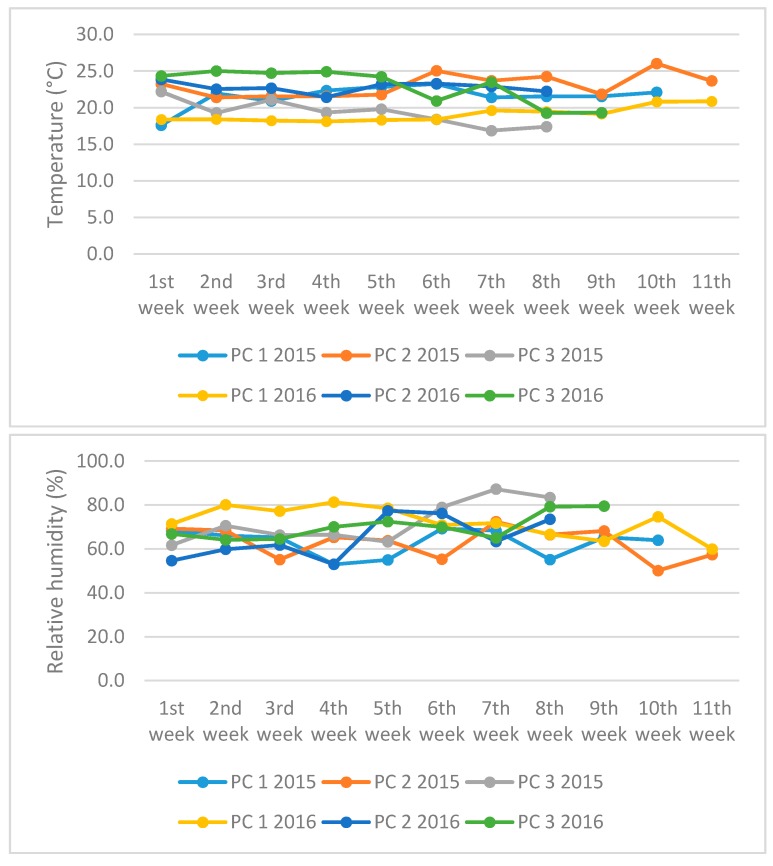

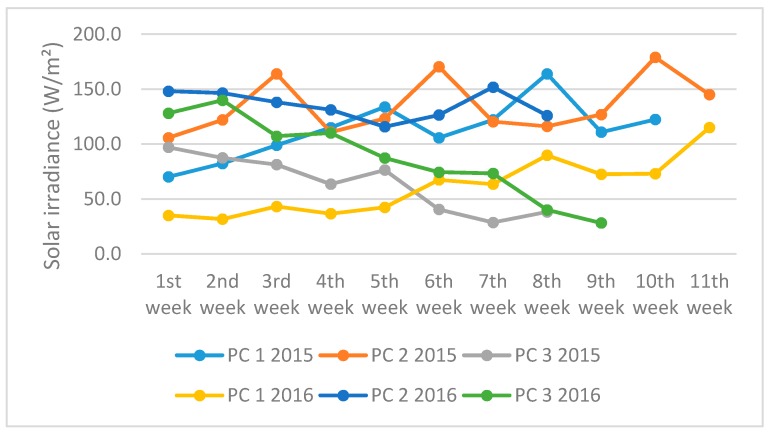
Cultivation conditions for three basil pot cultures in the greenhouse of FH MENDELU during the years 2015–2016. Upper graph: changes in average temperatures. Middle graph: changes in relative humidity. Lower graph: changes in solar irradiance. The presented pot cultures (PC) correspond to the following seasons: PC 1 2015 (23.3.–1.6.), PC 1 2016 (8.2.–25.4.), PC 2 2015 (27.4.–14.7.), PC 2 2016 (28.4.–20.6.), PC 3 2015 (1.9.–26.10.), and PC 3 2016 (15.8.–17.10.). For the plant material and cultivation conditions see Section 3.2.

Particular components of basil essential oils, identified by means of Thermo Finnigan NIST 02 Libraries software, are listed in Table 2. The data in Table 2 illustrate the relative amounts of the individual components detected in the sample profiles (expressed as mean values and corresponding standard deviations obtained from three parallel measurements). The data indicate that the samples’ profiles (i.e., basil essential oil components and their relative amounts) are differing from each other depending not only on variety, but also on cultivation conditions. The new variety, ‘Mánes’, exhibited significant relative amounts of methyl eugenol and eugenol in the spring and summer periods while the majority of eugenol was transformed to methyl eugenol in the autumn period. On the other hand, in case of eucalyptol, β-linalool, and α-bergamotenen, it was the opposite. When considering changes in these periods (see Figure 2), for example the decrease in solar irradiation and temperature and the increase in relative humidity during autumn period, these factors are supposed to be responsible for biotransformation processes leading to changes in the composition of basil essential oils. Analogically it was found out also for most of the other tested basil varieties. ‘Ohře’ exhibited significant relative amounts of eucalyptol, β-linalool, methyl eugenol, and δ-cadinene in the spring and summer periods while β-linalool and methyl eugenol dominated in autumn period. ‘Dark Green’ exhibited significant relative amounts of eucalyptol, β-linalool, α-bergamotenen, p-allylanisol, methyl eugenol, and eugenol in the spring and summer periods, while α-bergamotenen, p-allylanisol, and methyl eugenol dominated in autumn period. ‘Mammolo Genovese’ exhibited significant relative amounts of eucalyptol, α-bergamotenen, methyl eugenol, and eugenol in the spring and summer periods while α-bergamotenen and methyl eugenol dominated in the autumn period. In comparison with these varieties, ‘Lettuce Leaf’ exhibited significant relative amounts of eucalyptol, β-linalool, methyl eugenol, and δ-cadinene over all the seasons with higher or lower concentration variability (i.e., qualitative profile was relatively stable) while ‘Purple Opaal’ or ‘Red Rubin’ exhibited significant differences in qualitative and quantitative composition of major essential oil components such as eucalyptol, β-linalool, methyl eugenol, eugenol, and δ-cadinene or eucalyptol, α-bergamotenen, methyl eugenol, and eugenol in each season (i.e., qualitative profiles were relatively unstable and more depended on little seasonal changes). Based on the essential oil profiles and corresponding relative detection responses for particular essential oil components (the highest analytical signals), the ‘Red Rubin’, ‘Mammolo Genovese’, ‘Purple Opaal’, ‘Manes’, and ‘Dark Green’ varieties were identified as methyleugenol-rich, while ‘Ohře’ exhibited a variable chemotype depending on the season of cultivation (linalool/methyleugenol). ‘Lettuce Leaf’ was the sole sample with the estragole domination and it and ‘Dark Green’ were the only varieties containing detectable amounts of estragole.

### 2.3. Determination of Selected Essential Oil Components in Ocimum Basilicum Varieties

The essential oil components selected for the quantitative evaluation of the seven basil varieties were eugenol, β-linalool, eucalyptol, and estragole due to their general importance in the pharmaceutical, food, and cosmetic industries. Statistical evaluation of the GC-MS method employed for the quantitation included parameters of calibration lines (linear range, determination coefficient, slope, interface), detection and quantitation limits (LOD and LOQ, respectively), and reproducibility of measurements (standard deviation). The resulting performance and calibration parameters are summarized in Table 3. The data indicated that the quantitation limits, linear ranges, and reproducibility of the measurements were acceptable for sensitive and highly reliable determination of the tested components in basil essential oils extracted from seven studied basil varieties.

The application data in Table 4 illustrate concentrations of the selected components found in the tested essential oils. The concentrations are expressed as mean values and corresponding standard deviations obtained from three parallel measurements. The data indicate that the quantities of examined basil essential oil components differ from each other depending not only on variety but also on cultivation conditions.

Generally, the pot cultures 1 and 2 (covering the sunny season from half of March to half of July with higher temperature and lower relative humidity) exhibited significantly higher concentrations of the particular quantified essential oil components than pot culture 3 (covering darker season including September and October with lower temperature and higher relative humidity), see Table 4. Here, methyl eugenol, which exhibited opposite behavior, was an exception (changes in solar irradiation, temperature, and relative humidity were supposed to be responsible for increased biotransformation of eugenol to less preferred methyl eugenol in darker season, see Table 2). The concentrations of the quantified components in particular essential oils did not differ significantly when comparing pot cultures 1 and 2. From these observations it is clear that the controlled solar irradiation, temperature, and relative humidity are important factors for providing stable cultivation and, by that, production, or for enhancing production of eugenol, linalool, eucalyptol, and estragole as the key components of essential oils in basil varieties, or for minimizing production of less preferred/unwanted components (e.g., methyl eugenol). The new variety, ‘Mánes’, exhibited significant amounts of three essential oil components, namely eucalyptol, linalool, and eugenol, while estragole was not quantified, see concentration data in Table 4. This pattern was more or less similar to other examined basil varieties with exception for ‘Lettuce leaf‘ and ‘Dark green‘ in which estragole could also be quantified. When considering superior extraction yield of ‘Mánes’, the produced quantity of these important essential oil components is comparable or even better than ‘Purple Opaal’, ‘Mammolo Genovese’, ‘Red Rubin’, ‘Ohře’ (with exception of linalool content), and ‘Lettuce Leaf’ and ‘Dark Green’ (both with exception of estragole content) varieties.

## 3. Materials and Methods

### 3.1. Chemicals and Samples

Eugenol, linalool, eucalyptol, and estragole were purchased as reference standards from Sigma-Aldrich (Steinheim, Germany). All the chemicals used were of analytical grade. n-Hexane for gas chromatography was obtained from Merck (Darmstadt, Germany).

### 3.2. Plant Material and Cultivation Conditions

Seven basil varieties, namely ‘Ohře’, ‘Lettuce Leaf’, ‘Purple Opaal’, ‘Dark Green’, ‘Mammolo Genovese’, ‘Mánes’, and ‘Red Rubin’, were used in the experiments. The varieties were obtained from Semo Ltd. (Smržice, Czech Republic) and from Seva-Seed Ltd. (Valtice, Czech Republic).

Basil plants were cultivated as a pot culture in a greenhouse of the Mendel University in Brno, Faculty of Horticulture in Lednice.

Conditions for growing basil (temperature, humidity, slide shades) were controlled by the control system LCC 1240 (DGT Volmatic, Odense, Denmark). The control was ensured by PC SuperLink 4.0 (DGT Volmatic, Odense, Denmark) with a graphic visualization and archiving capabilities. The average temperature, relative humidity, and solar irradiance in each pot culture during the years 2015–2016 were divided into individual growing weeks and are illustrated in Figure 2.

The experimental layout included three individual sowing and harvesting periods (pot cultures 1–3) of the sweet basil in one calendar year. These were following: pot culture 1 (23.3.–1.6. 2015 and 8.2.–25.4. 2016), pot culture 2 (27.4.–14.7. 2015 and 28.4.–20.6. 2016), pot culture 3 (1.9.–26.10. 2015 and 15.8.–17.10. 2016). The greenhouse experiments were carried out in 4 replications for each variety with 30 plants for each variant.

Basil was grown in 500 mL plastic pots, at 20 plants per pot. The plants were cultured in medium Horticultural substrate B with active humus (Rašelina Soběslav Ltd., Soběslav, Czech Republic), irrigated as needed, protected from pests and diseases, and once fertilized (Kristalon Gold, Agro CS, Lučenec, Slovak Republic; dosage: 10 g of fertilizer per 10 L of water). The basil plants were harvested before their flowering. After drying (at room temperature) the plants were stored in paper bags in a dark place until the time of steam distillation.

### 3.3. Steam Distillation of Basil Essential Oil

The content of the basil essential oil in the aerial part of plant was determined at the Department of Vegetable Growing and Floriculture of the Mendel University in Brno by the method of steam distillation according to [21], without using xylene. The samples of plant material (dried leaf and stem tissue) were fragmented in a laboratory mill (ILABO MF 10 basic, with maximum size of grain 3.15 mm) immediately before the distillation. An amount of 30.0 g of the crushed drug was added in a 1000 mL flask; the content of distillation liquid (water) was 400 mL. The distillation was carried out at a rate of 2–3 mL·min^−1^ for 2 h. The volume of isolated essential oil was measured in a proper calibrated tube. The extracted essential oils were stored in sealed vials at 4 °C in the refrigerator until GC-MS analysis. The content of essential oils in the plant material was calculated from 2 parallel distillations and expressed in mL·kg^−1^.

### 3.4. Apparatus

Gas chromatography analyzer Trace GC, Thermo Finnigan (Waltham, MA, USA) with autosampler AS 2000 (Thermo Finnigan, Waltham, MA, USA) was used for the analysis of the extracted basil essential oil samples. Mass spectrometry detector Trace DSQ (Thermo Finnigan, Waltham, MA, USA) was an integral part of the gas chromatographic analyzer. The GC-MS records were processed by means of Thermo Finnigan Xcalibur 1.3 software (Thermo Finnigan, Waltham, MA, USA). The GC-MS instrumental parameters and conditions are given in Section 3.5 GC-MS conditions.

The distillation apparatus Gerhardt (Königswinter, Germany) was used for the extraction of essential oils from the basil plant material. Distilled water used in the experiments was prepared by an Aqua Osmotic type 02 (Aqua Osmotic, Tišnov, Czech Republic) water purification system. The distilled water meets the standards according to ČSN 68 4063, Czech Pharmacopoeia 2005, and ČSN ISO 3696.

### 3.5. GC-MS Conditions

The identity and quantity of particular components of the basil essential oil were evaluated by the GC-MS analysis method, using gas chromatograph Trace GC equipped with MS detector Trace DSQ and DB-WAX (highly polar capillary column with 100% polyethylene glycol stationary phase) capillary column (circa 30 m of length with inner diameter of 0.25 mm and a 0.25 μm layer of the inner film). The measurement records started 3 min after the run beginning. Temperature of the GC column was kept at 40 °C for 3 min. Then, the temperature gradually increased with the gradients of 8 °C/min up to 60 °C, 5 °C/min up to 70 °C, and 4 °C/min up to 230 °C. The temperature of 230 °C was kept constant for 1 minute. Subsequent GC working conditions were as follows: carrier gas was He with a constant flow rate of 0.5 mL/min, matrix solvent was n-hexane. MS working conditions were as follows: temperature of ion source was 200 °C, temperature MS transfer line was 200 °C, mass range was 20–300 *m*/*z*. Injection conditions were as follows: 10 μL injection syringe, injection volume 1 μL, injection temperature was 240 °C, regime of injection was “split”, split flow was 50 mL/min, split ratio was 100. The content of selected components of the essential oils was calculated from 3 parallel analyses and expressed in mg/mL.

### 3.6. Qualitative, Quantitative, and Statistical Evaluation of Analytical Data

Thermo Finnigan NIST 02 Libraries software (Thermo Finnigan, Waltham, MA, USA) was applied to the identification of particular components of basil essential oil. Parameters of calibration lines of the selected components (eugenol, linalool, eucalyptol, estragole) present in the basil essential oils were calculated by using Microsoft Excel 2007 (Microsoft Corporation, Redmond, Washington, WA, USA). The program Statistica Cz v. 12 (StatSoft, www.statsoft.cz) was used for the statistical evaluation of the results.

## 4. Conclusions

It can be summarized that both variety and season of cultivation have a significant effect on (i) the content (extraction yield) and the (ii) qualitative as well as (iii) quantitative profile of basil essential oils. Chemotypes of the studied varieties were evaluated by quantification of four main basil essential oil constituents (i.e., eucalyptol, linalool, estragole, eugenol) and comparing the obtained results with data presented in the literature. According to these criteria, ‘Ohře’, ‘Lettuce Leaf’, ‘Purple Opaal’, and ‘Dark Green’ were identified as linalool chemotypes, which was in good agreement with the results by Beatovic et al. [11], Klimankova et al. [12], and Svecova [14]. Although linalool is declared to be the dominant component of the oil derived from European basil varieties [11,13,15,16], ‘Mammolo Genovese’, ‘Mánes’, and ‘Red Rubin’, studied as pot cultures in our work, were identified rather as eucalyptol chemotypes. Anyway, a significant amount of linalool in ‘Mánes’, and 'Red Rubin' corresponded well with the general characteristic features of European basil varieties. In detailed analysis, mixed chemotypes (i.e., with similar concentrations of main quantified essential oil constituents) were found for ‘Lettuce Leaf’ (linalool:estragole, 1.0:0.9), ‘Dark Green’ (linalool:eucalyptol, 1.0:0.7), ‘Mammolo Genovese’ (eucalyptol:eugenol, 1.0:0.9), ‘Mánes’ (eucalyptol:linalool:eugenol, 1.0:0.6:0.4), and ‘Red Rubin’ (eucalyptol:linalool:eugenol, 1.0:0.6:0.7). Moreover, when considering also other compounds identified in the GC-MS profiles, all the studied varieties, except ‘Lettuce Leaf’, were methyl eugenol-rich with strong dependence of the eugenol:methyl eugenol ratio on seasonal changes (solar irradiation, temperature, relative humidity). Similarly to our results, Vani et al. [22], based on GC analysis, confirmed the higher values of methyl eugenol in September and October. These findings suggested that biotransformation in the basil varieties can be effectively manipulated by controlling cultivation conditions. In this way, maximization/minimization of the amount of target component in essential oil can be provided effectively, not only by the selection of appropriate basil variety, but also by the selection of appropriate pot culture (season). For example, the production of important components of the basil essential oils such as eucalyptol, linalool, estragole, and eugenol was more or less pronouncedly enhanced by increasing solar irradiation and temperature while production of less-preferred methyl eugenol was decreased under these conditions.

Surprisingly, the new variety, ‘Mánes’, exhibited considerably higher extraction yield when compared to other examined basil varieties (‘Ohře’, ‘Lettuce Leaf’, ‘Purple Opaal’, ‘Dark Green’, ‘Mammolo Genovese’, and ‘Red Rubin’). Here, the extraction yield was enhanced two to five times depending on the compared variety. When considering the superior and stable (i.e., reproducible in inter-seasonal or inter-year measure) extraction yield and favorable essential oil profile of ‘Mánes’ towards the generally important essential oil components, this basil variety is very promising for use in the pharmaceutical, food, and cosmetic industries.

## Figures and Tables

**Table 1:** Yield of *Ocimum basilicum* essential oil (mL·kg^−1^) d. w.

Variety	2015				2016			
	PC 1	PC 2	PC 3	Mean *	PC 1	PC 2	PC 3	Mean *
‘Ohře’	7.7	8.5	8.3	8.2 ± 0.9 ^cA^	6.8	8.3	7.2	7.4 ± 0.7 ^cA^
‘Lettuce Leaf’	4.8	5.0	4.8	4.9 ± 0.3 ^abB^	3.9	4.0	3.9	3.9 ± 0.2 ^aA^
‘Purple Opaal’	5.5	5.5	4.0	5.0 ± 0.8 ^abA^	3.4	5.1	3.6	4.1 ± 0.8 ^aA^
‘Dark Green’	6.6	5.7	5.4	5.9 ± 0.8 ^bB^	3.9	5.3	4.4	4.5 ± 0.6 ^abA^
‘Mammolo Genovese’	7.0	8.9	8.6	8.1 ± 1.0 ^cB^	6.0	5.5	4.3	5.3 ± 0.8 ^bA^
‘Mánes’	17.5	17.1	17.2	17.3 ± 0.2 ^dA^	17.5	18.1	17.4	17.7 ± 0.4 ^dA^
‘Red Rubin’	4.4	4.6	3.1	4.0 ± 0.8 ^aA^	3.9	3.4	3.8	3.7 ± 0.3 ^aA^

Note: PC 1—Pot culture 1; PC 2—Pot culture 2; PC 3—Pot culture 3; * Values are mean ± SD of three different pot cultures of the *Ocimum basilicum* varieties, distilled (Section 3.3) individually in two parallels. Mean with the same lowercase letters in a column and uppercase letters on a line do not differ at 95% by Tukey's test.

**Table 2:** Identified components and their relative contents in essential oils of seven varieties of *Ocimum basilicum* L ^a^.

Variety	‘Ohře’			‘Lettuce Leaf’			‘Purple Opaal’			‘Dark Green’			‘Mammolo Genovese’			‘Mánes’			‘Red Rubin’		
Pot Culture	1	2	3	1	2	3	1	2	3	1	2	3	1	2	3	1	2	3	1	2	3
Eucalyptol (%) SD	5.94	8.88	3.44	3.64	4.12	2.18	4.05	5.50	1.45	7.09	11.70	4.34	9.56	11.59	3.56	4.33	4.74	1.78	3.33	4.92	3.48
	0.457	0.370	0.187	0.137	0.122	0.076	0.111	0.657	0.031	0.927	0.025	0.276	0.074	0.015	0.044	0.148	0.113	0.033	0.192	0.401	1.027
Fenchone (%) SD	1.19	1.33	1.76	0.00	0.00	0.00	0.99	1.12	1.10	0.00	0.00	0.00	0.00	0.00	0.00	0.00	0.00	0.00	0.36	0.42	0.51
	0.061	0.085	0.071	0.000	0.000	0.000	0.015	0.123	0.147	0.000	0.000	0.000	0.000	0.000	0.000	0.000	0.000	0.000	0.056	0.006	0.153
Fenchyl acetate (%) SD	1.03	0.66	1.28	0.00	0.00	0.00	0.97	0.59	1.88	0.00	0.00	0.00	0.00	0.00	0.00	0.00	0.00	0.00	0.25	0.39	0.37
	0.071	0.091	0.056	0.000	0.000	0.000	0.047	0.015	0.071	0.000	0.000	0.000	0.000	0.000	0.000	0.000	0.000	0.000	0.025	0.060	0.085
β-Linalool (%) SD	33.47	31.29	22.74	10.50	6.57	8.55	10.75	11.59	3.67	8.90	10.28	1.19	1.26	1.34	0.21	2.60	2.08	0.43	1.73	2.69	1.86
	2.005	0.751	0.591	0.310	0.367	0.050	0.169	0.132	0.153	0.610	0.285	0.081	0.331	0.189	0.076	0.052	0.025	0.070	0.146	0.142	0.594
α-Bergamotene (%) SD	0.00	0.54	0.04	8.65	10.78	9.98	0.00	0.52	0.00	10.71	11.50	7.87	13.38	13.11	8.73	4.36	5.24	2.04	9.69	10.50	9.15
	0.000	0.545	0.010	0.042	0.347	0.187	0.000	0.006	0.000	0.168	0.046	0.545	0.223	0.868	0.128	0.025	0.340	0.058	0.040	1.974	0.188
Caryophyllene (%) SD	2.91	1.34	1.20	0.00	0.00	0.00	1.61	0.78	0.92	0.00	0.00	0.00	0.00	0.00	0.00	0.70	1.76	0.68	0.00	0.00	0.00
	0.866	0.284	0.035	0.000	0.000	0.000	0.040	0.121	0.021	0.000	0.000	0.000	0.000	0.000	0.000	0.005	0.125	0.148	0.000	0.000	0.000
Isocaryophyllene (%) SD	1.89	2.06	2.05	0.00	0.11	0.19	2.54	2.82	2.63	0.00	0.08	0.07	0.00	0.14	0.22	0.83	0.00	0.00	0.10	0.50	0.26
	0.311	0.066	0.046	0.000	0.006	0.035	0.026	0.015	0.300	0.000	0.006	0.010	0.000	0.017	0.046	0.025	0.000	0.000	0.031	0.295	0.006
4-Carvomenthol (%) SD	0.00	0.00	0.00	1.60	1.81	0.88	0.00	0.00	0.00	0.25	0.15	0.00	0.00	0.00	0.00	0.00	0.00	0.00	0.00	0.00	0.00
	0.000	0.000	0.000	0.045	0.051	0.122	0.000	0.000	0.000	0.026	0.006	0.000	0.000	0.000	0.000	0.000	0.000	0.000	0.000	0.000	0.000
β-Farnesene (%) SD	0.71	0.46	1.56	0.51	0.35	0.76	2.65	1.36	2.25	2.10	1.04	3.18	3.08	1.76	2.84	5.05	2.70	3.81	3.23	1.82	2.17
	0.075	0.107	0.010	0.006	0.015	0.040	0.036	0.081	0.314	0.010	0.015	0.051	0.103	0.050	0.040	0.005	0.108	0.009	0.471	0.153	0.410
α-Caryphyllene (%) SD	1.59	2.27	1.43	0.00	0.00	0.00	1.15	1.26	1.19	0.00	0.00	0.00	1.57	1.95	1.49	0.46	0.62	0.46	0.67	0.97	1.01
	0.106	0.187	0.010	0.000	0.000	0.000	0.091	0.026	0.147	0.000	0.000	0.000	0.061	0.188	0.061	0.037	0.050	0.005	0.036	0.217	0.070
Estragole (%) SD	0.00	0.00	0.00	42.56	48.28	39.16	0.00	0.00	0.00	6.85	5.14	4.60	0.00	0.00	0.00	0.00	0.00	0.00	0.00	0.00	0.00
	0.000	0.000	0.000	1.077	1.573	0.086	0.000	0.000	0.000	1.650	0.025	1.130	0.000	0.000	0.000	0.000	0.000	0.000	0.000	0.000	0.000
β-Cubebene (%) SD	2.80	2.33	0.91	3.32	2.70	3.90	1.88	1.74	0.90	2.04	1.80	1.25	1.21	0.99	0.69	1.22	1.08	0.49	0.82	0.92	0.58
	0.197	0.242	0.006	0.071	0.159	0.086	0.036	0.157	0.081	0.015	0.025	0.238	0.046	0.110	0.026	0.012	0.017	0.029	0.081	0.047	0.055
α-Bulnesene (%) SD	1.32	0.61	0.98	0.61	0.25	0.92	0.58	0.29	0.41	0.46	0.30	0.27	0.27	0.24	0.09	0.34	0.26	0.08	0.26	0.29	0.10
	0.070	0.117	0.015	0.045	0.021	0.006	0.032	0.036	0.040	0.012	0.040	0.056	0.000	0.046	0.000	0.008	0.005	0.012	0.046	0.046	0.012
γ-Cadinene (%) SD	4.26	3.46	1.66	3.10	2.23	3.32	2.79	2.29	1.56	2.04	1.64	0.97	0.78	0.63	0.43	0.30	0.40	0.10	0.61	0.81	0.46
	0.248	0.396	0.021	0.172	0.096	0.203	0.056	0.198	0.067	0.046	0.026	0.335	0.050	0.086	0.060	0.008	0.005	0.012	0.036	0.066	0.006
trans-Geraniol (%) SD	4.62	2.37	1.46	0.29	0.00	0.00	0.46	0.34	0.00	0.00	0.00	0.00	0.00	0.00	0.00	0.00	0.00	0.00	0.00	0.00	0.00
	0.118	0.010	0.075	0.031	0.000	0.000	0.101	0.061	0.000	0.000	0.000	0.000	0.000	0.000	0.000	0.000	0.000	0.000	0.000	0.000	0.000
Methyl eugenol (%) SD	17.61	19.94	42.17	10.61	9.24	15.45	54.60	55.49	50.30	35.07	27.96	68.18	36.06	28.12	71.89	69.20	66.49	87.04	60.95	59.04	71.23
	0.246	3.062	0.229	0.192	0.025	1.072	0.500	0.626	3.115	1.214	0.339	2.786	1.335	1.743	0.675	0.380	2.201	0.359	1.718	4.543	1.184
Eugenol (%) SD	8.19	11.45	2.02	1.59	3.38	0.00	3.71	4.83	0.83	14.50	18.81	2.18	26.01	33.50	4.67	7.74	11.81	0.74	14.80	12.27	3.67
	0.056	0.050	0.136	0.137	0.091	0.000	0.626	0.625	0.006	0.692	0.253	0.110	1.082	0.312	0.214	0.185	1.850	0.012	1.990	3.237	0.070
δ-Cadinene (%) SD	6.67	5.60	1.40	7.39	5.49	8.86	5.82	4.98	2.06	4.29	3.48	2.16	0.00	0.00	0.78	0.00	0.00	0.26	0.00	0.00	1.26
	0.607	0.986	0.130	0.152	0.396	0.417	0.025	0.504	0.252	0.272	0.015	0.531	0.000	0.000	0.198	0.000	0.000	0.046	0.000	0.000	0.059

^a^ Conditions for the sample preparation (cultivation, extraction) and GC-MS analysis are in the Section 3. Materials and methods. Pot cultures from the year 2015 are illustrated in the present table.

**Table 3:** Performance parameters and calibration data for determined basil essential oil components analyzed by the GC-MS method.

Parameter	Eucalyptol	β-Linalool	Estragole	Eugenol
LOD (mg/mL)	0.0076	0.0022	0.0085	0.0063
LOQ (mg/mL)	0.0087	0.0090	0.0118	0.0066
Linear range (mg/mL)	0.0184–0.1105; 0.1840–1.8420; 0.5530–3.6840	0.0087–2.6100; 0.8700–8.7000	0.0097–0.4825; 0.0965–4.8250	0.0107–0.1067; 0.1067–3.2010
Slope	8,000,000; 5,000,000; 10,000,000	4,000,000; 4,000,000	20,000,000; 20,000,000	20,000,000; 20,000,000
SD(slope)	263,938; 177,592; 543,176	103,200; 70,559	590,333; 516,332	304,140; 244,730
Intercept	−56,416; −558,645; −4,000,000	3151; −901,728	−141,065; 349,731	−124,428; −163,394
SD(intercept)	17,973; 215,782; 1,314,102	142,033; 356,621	145,963; 1,276,657	19,651; 353,406
Determination coefficient	0.9989; 0.9976; 0.9947	0.9984; 0.9994	0.9985; 0.9985	0.9996; 0.9992

**Table 4:** Quantified components in essential oils of seven varieties of *Ocimum basilicum* L ^a^.

Variety	‘Ohře’			‘Lettuce Leaf’			‘Purple Opaal’			‘Dark Green’			‘Mammolo Genovese’			‘Mánes’			‘Red Rubin’		
Pot Culture	1	2	3	1	2	3	1	2	3	1	2	3	1	2	3	1	2	3	1	2	3
Eucalyptol (mg/mL) SD	1.26	1.67	0.38	0.95	0.99	0.52	1.12	1.28	0.04	1.55	1.52	1.00	1.59	1.54	0.88	1.22	1.14	0.51	0.87	1.03	0.37
	0.051	0.107	0.021	0.021	0.056	0.006	0.051	0.132	0.008	0.132	0.086	0.042	0.010	0.006	0.015	0.075	0.026	0.006	0.015	0.071	0.572
β-Linalool (mg/mL) SD	8.40	7.13	2.45	3.26	1.98	2.22	3.53	3.30	0.20	3.03	2.70	0.30	0.39	0.34	0.05	1.06	0.56	0.12	0.51	0.63	0.24
	0.255	0.021	0.021	0.117	0.155	0.036	0.032	0.021	0.051	0.190	0.250	0.021	0.096	0.056	0.021	0.036	0.006	0.024	0.070	0.026	0.337
Estragole (mg/mL) SD	0.00	0.00	0.00	2.44	2.55	1.81	0.00	0.00	0.00	0.43	0.23	0.21	0.00	0.00	0.00	0.00	0.00	0.00	0.00	0.00	0.00
	0.000	0.000	0.000	0.040	0.006	0.047	0.000	0.000	0.000	0.107	0.021	0.061	0.000	0.000	0.000	0.000	0.000	0.000	0.000	0.000	0.000
Eugenol (mg/mL) SD	0.41	0.51	0.04	0.10	0.19	0.00	0.23	0.26	0.01	0.92	0.90	0.12	1.62	1.70	0.25	0.50	0.64	0.05	0.84	0.58	0.27
	0.010	0.015	0.002	0.010	0.006	0.000	0.031	0.031	0.002	0.046	0.059	0.006	0.042	0.031	0.015	0.026	0.122	0.001	0.082	0.152	0.272

^a^ Conditions for the sample preparation (cultivation, extraction) and GC-MS analysis are in the Section 3. Materials and methods. Pot cultures from the year 2015 are illustrated in the present table.
